# Interactive relationships of Type 2 diabetes and bipolar disorder with cognition: evidence of putative premature cognitive ageing in the UK Biobank Cohort

**DOI:** 10.1038/s41386-022-01471-6

**Published:** 2022-10-15

**Authors:** Elysha Ringin, David W. Dunstan, Roger S. McIntyre, Michael Berk, Neville Owen, Susan L. Rossell, Tamsyn E. Van Rheenen

**Affiliations:** 1 Melbourne Neuropsychiatry Centre, Department of Psychiatry, University of Melbourne and Melbourne Health, Melbourne, VIC Australia; 2 Baker Heart & Diabetes Institute, Melbourne, VIC Australia; 3 Institute for Physical Activity and Nutrition (IPAN), School of Exercise and Nutrition Sciences, Deakin University, Geelong, VIC Australia; 4 Mood Disorders Psychopharmacology Unit, University of Toronto, Toronto, ON Canada; 5 Deakin University, The Institute for Mental and Physical Health and Clinical Translation, Barwon Health, Geelong, VIC Australia; 6 Department of Psychiatry, University of Melbourne, Melbourne, VIC Australia; 7 Orygen Youth Health, Melbourne, VIC Australia; 8 Centre for Urban Transitions, Swinburne University of Technology, Melbourne, VIC Australia; 9 Centre for Mental Health, School of Health Sciences, Swinburne University, Melbourne, VIC Australia; 10 St Vincent’s Mental Health, St Vincent’s Hospital, Melbourne, VIC Australia

**Keywords:** Cognitive ageing, Cognitive neuroscience, Psychology, Bipolar disorder

## Abstract

Type 2 diabetes (T2D) is disproportionately prevalent in bipolar disorder (BD) and is associated with cognitive deficits in psychiatrically healthy cohorts. Whether there is an interaction effect between T2D and BD on cognition remains unclear. Using the UK Biobank, we explored interactions between T2D, BD and cognition during mid and later life; and examined age-related cognitive performance effects in BD as a function of T2D. Data were available for 1511 participants with BD (85 T2D), and 81,162 psychiatrically healthy comparisons (HC) (3430 T2D). BD and T2D status were determined by validated measures created specifically for the UK Biobank. Diagnostic and age-related associations between T2D status and cognition were tested using analyses of covariance or logistic regression. There was a negative association of T2D with visuospatial memory that was *specific* to BD. Processing speed and prospective memory performance were negatively associated with T2D, irrespective of BD diagnosis. Cognitive deficits were evident in BD patients *with* T2D compared to those without, with scores either remaining the same (processing speed) or improving (visuospatial memory) as a function of participant age. In contrast, cognitive performance in BD patients *without* T2D was worse as participant age increased, although the age-related trajectory remained broadly equivalent to the HC group. BD and T2D associated with cognitive performance deficits across the mid-life period; indicating comorbid T2D as a potential risk factor for cognitive dysfunction in BD. In comparison to BD participants without T2D *and* HCs, age-independent cognitive impairments in BD participants with comorbid T2D suggest a potential premature deterioration of cognitive functioning compared to what would normally be expected.

## Introduction

Bipolar disorder (BD) is associated with substantial cognitive heterogeneity, where more than 50% of patients experience cognitive dysfunction that persists during euthymic phases and contributes significantly to psychosocial burden [1–5]. Although the drivers of this heterogeneity remain unclear [6, 7], physical health has become increasingly recognised as an important consideration in BD outcomes—including cognition—due to the higher prevalence and earlier onset of age-related medical conditions in people with the illness [8]. These conditions may index a premature biological ageing process in BD, which influences neuroprogression and somatoprogression [9], and is driven by inflammation, oxidative stress, telomere shortening, and altered neurotrophin levels, brain structure and function [10]. In the general population, biological changes of this type are associated with older age and cognitive decline [11]. Thus, premature cognitive ageing (i.e., the presence of cognitive impairments earlier than expected) may represent one potential explanation for cognitive dysfunction in BD [12–14].

One age-related medical condition commonly seen in BD is Type 2 diabetes (T2D), with a prevalence of 11% (7.9% in the general population) [15, 16], and a two-fold risk of development compared to psychiatrically healthy people [17]. Several pathophysiological and behavioural factors that are linked to cognitive dysfunction are also evident in T2D and BD, including unhealthy lifestyle choices such as poor diet or lack of exercise, and changes in blood lipids, inflammation, oxidative stress, mitochondrial dysfunction, and HPA axis dysregulation [10, 18–21]. Relevantly, T2D is associated with poorer cognition in the general population [22, 23], and, similar to BD, increases the risk of age-related cognitive disorders including all-cause dementia, vascular dementia, and Alzheimer’s disease [22, 24]. Cognitive deficits implicated in T2D include those in the domains of memory, attention, processing speed, language, perception/construction, executive function, and global cognition [22, 23]. These domains are also consistently impaired in BD [25, 26], with processing speed and executive function seemingly affected earlier in the lifespan than would be expected on account of age-related decline [12–14].

Since T2D and its precursor, metabolic syndrome, present in some patients with BD at an earlier age than usual [27, 28], it is plausible that the presence and extent of cognitive dysfunction in BD are related to the presence of T2D. However, whether this is true remains unknown, since few BD studies have explored cognitive functioning and T2D or even its component factors [29–32]. Of the available evidence, two past studies reported no evidence of a relationship between glucose and cognition, however, these studies were limited by the primarily normoglycemic sample [29, 30]. A separate study found no association between diabetes medication use and cognition in a combined BD and schizophrenia sample [31]. In contrast, a negative association between diabetes and general cognition was observed in a study of older BD patients (>60 years) [32], while we recently reported preliminary findings linking triglyceride levels (associated with insulin resistance as a precursor to T2D) to reduced cognitive flexibility in adults with BD [33]. Given these mixed findings and the paucity of cognitive BD studies including T2D diagnosis explicitly as a factor of interest, it is not clear whether comorbid T2D contributes to cognitive dysfunction in BD, and if so, whether it is associated with cognitive dysfunction at an earlier point in the lifespan than would be expected on account of normal age-related cognitive decline.

Herein, we compared the cognitive performance of BD patients with and without T2D to a psychiatrically healthy comparison (HC) group using a large cross-sectional sample derived from the UK Biobank (UKB), to examine whether cognitive performance was moderated by the presence of T2D. Specifically, we used a quasi-longitudinal design to examine associations between BD, T2D and cognition as a function of participant age. We hypothesised that worse cognitive performance would be evident in the BD and HC groups with T2D compared to those without, and that these differences would be more pronounced in those with BD. We also expected those with BD and comorbid T2D would have worse cognitive performance compared to other groups, irrespective of age.

## Materials and methods

The UKB is a prospective dataset of 502,649 participants, aged 40–69. Baseline assessments were completed across 22 centres throughout the UK between 2005 and 2010, providing a range of lifestyle, health, demographic, cognitive, and biological data. Full details of the data collection procedures are provided elsewhere [34]. All participants provided written informed consent. The UKB has approval from the Northwest Multi-Centre Research Ethics committee (reference 16/NW/0274 and 11/NW/0382).

### BD diagnostic criteria

UKB participants categorised as either having *probable* BD or as being psychiatrically healthy were selected for this analysis. The full methodology of categorising participants is detailed elsewhere [35]. In brief, a touchscreen questionnaire based on symptoms within the Structured Clinical Interview for DSM-IV axis I disorders (introduced in the final two years of recruitment) was utilised to identify participants with probable BD, major depressive disorder, or no indicated mental disorder, which was validated against demographic and clinical information available in the dataset. For readability and to remain consistent with previous studies using UKB data [36, 37], participants identified as having probable BD are henceforth referred to as simply having ‘BD’. In our analyses, participants with any mental disorder, or using any type of psychotropic medication, were excluded from the HC group. Participants who were pregnant, as well as those with neurological conditions known to affect cognitive functioning, were excluded from both the BD and HC groups (see supplementary).

### Diabetes mellitus prevalence

An algorithm by Eastwood et al. [38] was used to determine the prevalence of T2D in the current sample. The algorithm was validated against primary and secondary medical records in a subset of UKB participants (sensitivity rate of 96%). In brief, the algorithm uses medical history to categorise participants as having possible T2D, probable T2D, possible type 1 diabetes (T1D), probable T1D, gestational diabetes, or diabetes unlikely. As the focus of the current study was on T2D, participants categorised as having possible T2D, possible T1D, probable T1D, and gestational diabetes were excluded. Those with *probable T2D* were included, but for readability and to remain consistent with previous studies using UKB data [39, 40], probable T2D is henceforth simply be referred to as ‘T2D’.

### Participant characteristics (confounding factors)

Age, sex, educational level, and socio-economic status (SES)—measured by the Townsend Deprivation Index—were collected from questionnaires completed during baseline assessments. Education was dichotomously coded as participants either having a university/college degree or not. Waist circumference measurements were recorded by an on-site research assistant during physical health assessments, as detailed elsewhere [34]. Medication use was reported during the verbal interview. In the current study, participants were dichotomised according to whether or not they were taking different classes of psychotropic medication (i.e., mood stabilisers, antidepressants, first-generation antipsychotics, second-generation antipsychotics, and sedatives/hypnotics) and whether or not they were taking diabetes medications (appendices S1, S2).

### Cognitive assessment

Cognitive functioning was assessed through a brief computerised battery taking approximately 15 min to complete. The battery was developed specifically for the UKB and designed to be completed electronically without examiner supervision. Assessments were completed at the UKB assessment centres and included measurement of the four cognitive domains listed below (UKB test name in brackets) [A fifth cognitive domain, numeric memory, was tested at baseline. A recent publication has queried whether the numeric memory test designed for the UKB accurately tests its intended cognitive domain; working memory [41]. Further, the test was removed during the early stages of testing due to time constraints, resulting in a very low number of participants with available data. For these reasons, this domain is not used in the current study.]. Full details can be found elsewhere [42] [The UKB does not provide normed scores. Irrespective, raw scores were analysed given the presence of a control group and our capacity to control age and education directly in the analyses.].

1. Visuospatial memory (pairs matching): participants observed 6 sets of symbol cards which were then turned face down and asked to remember as many matching pairs as possible in the fewest tries. The dependent variable (DV) was the number of errors. Higher scores indicate worse performance.
2. Processing speed (reaction time): participants viewed pairs of cards and pressed a button when the cards matched. The DV was the mean response time (milliseconds). Higher scores indicate worse performance.
3. Reasoning (fluid intelligence): participants solved a number of numeric and verbal logic problems in 2 min. The DV was the number of correct problems solved. Higher scores indicate better performance.
4. Prospective memory: participants were given an instruction during the early stage of the cognitive testing, which they were asked to act on after a delay/distraction period. The DV was a dichotomously coded score indicating whether participants acted correctly or incorrectly in response to the instruction.

Note that reasoning was added towards the end of the recruitment period, and a number of participants skipped/abandoned certain tests. As such, participant numbers vary for each test in the analysis.

### Statistical analysis

All analyses were completed using the Statistical Package for the Social Sciences (SPSS) version 27 (IBM). Differences in demographic data between T2D and non-T2D participants in both diagnostic groups were assessed using χ^2^ tests and one-way ANOVAs.

ANCOVA and logistic regression were used to examine the associations between T2D and cognitive function [Information regarding additional analyses accounting for insulin use, HbA1c levels (indicative of insulin resistance), physical activity, and smoking status in the sample are included in the supplementary material. Given the absence of effects, they are presented alongside information about data cleaning.]. For the continuous cognitive variables (visuospatial memory, processing speed, and reasoning), multiple univariate ANCOVAs were used. Cognitive scores were specified as DVs, and T2D status, diagnostic group, and their interaction as the independent variables of interest. Age, sex, SES, waist circumference, and educational level, were selected as covariates a-priori. A T2D status *by* diagnostic group *by* age interaction term was also examined to determine whether the association of T2D on cognition was moderated by age in either diagnostic group. A logistic regression model was fitted for prospective memory (dichotomous DV). Independent variables and covariates remained the same. Covariates were added in block 1 of the model, T2D status and diagnostic group in block 2, and the interaction terms in block 3.

When significant three-way age interactions were found in either of the above-mentioned analyses, the models were stratified by diagnostic group and the T2D *by* age interaction term was tested again to determine age-related *diagnostic* slopes of cognitive performance. Here, cognitive performance in the BD and HC groups was modelled by age and T2D status, although in cases in which the T2D *by* age interaction effect was not significant, T2D subgroups within that group were collapsed (for ease of comparison).

Exploratory analyses were also conducted to examine associations between cognition and medication use in the sample (see Supplementary Material). A false discovery rate of *p* < 0.05 was applied to all results to account for multiple comparisons using the Benjamini–Hochberg method.

## Results

### Participants

Demographic characteristics of the study sample are displayed in Table 1. Sufficient data were available for 82,673 participants, including those for which the mood disorder screening, diabetes algorithm, and data for the covariates and at least one cognitive task were available. In total, 1511 participants met UKB criteria for BD (85 T2D, 1426 non-T2D) and 81,162 were HCs (3430 T2D, 77,732 non-T2D) (Figure S1).

**Table 1:** Characteristics of participants.

Characteristic	BD T2D (*n* = 85)	BD no T2D (*n* = 1426)	HC T2D (*n* = 3430)	HC no T2D (*n* = 77732)	Comparison	Post-hoc comparisons*
Age	57.7 ± 7.7	54.2 ± 8.01	60.8 ± 6.5	57.1 ± 8.1	F = 306.8, *p* < 0.001*	BD no T2D < all others, BD T2D < HC T2D, HC no T2D < HC T2D
Age diabetes diagnosed	50.74 ± 10.0		54.22 ± 10.0		F = 9.7, *p* = 0.002*	BD < HC
Sex (% male)	72.9	49.6	67.5	49.5	χ^2^ = 446.6, *p* < 0.001*	----
Waist circumference	105.4 ± 13.7	91.9 ± 14.2	102.2 ± 13.3	89.5 ± 12.9	F = 1111.4, *p* < 0.001*	BD T2D > both HCs, HC no T2D < all others, BD no T2D < HC T2D
Townsend deprivation index	0.5 ± 3.5	−0.003 ± 3.3	−0.6 ± 3.1	−1.4 ± 2.8	F = 212.2, *p* < 0.001*	BD T2D > BD no T2D and HC no T2D, BD no T2D > HC T2D and HC no T2D, HC T2D > HC no T2D
Educational level (% attended university)	28.2	40.0	26.2	34.5	χ^2^ = 106.3, *p* < 0.001*	----
Diabetes medication (% using)	62.4	----	69.6	----	χ^2^ = 2.07, *p* = 0.151	----
Mood stabilisers (% using)	14.1	12.0	----	----	χ^2^ = 0.34, *p* = 0.559	----
Antidepressants (% using)	49.1	26.0	----	----	χ^2^ = 7.27, *p* = 0.007*	----
First-generation antipsychotics (% using)	2.4	1.7	----	----	χ^2^ = 0.21, *p* = 0.645	----
Second-generation antipsychotics (% using)	7.1	5.7	----	----	χ^2^ = 0.28, *p* = 0.596	----
Sedatives/hypnotics (% using)	7.1	3.6	----	----	χ^2^ = 2.53, *p* = 0.112	----

*SSRIs* selective serotonin reuptake inhibitors. Note that higher Townsend deprivation index scores indicate lower socioeconomic status.

*Significant at *p* < 0.05

---- Data not applicable

Data are expressed as mean ± SD.

### Primary analyses

Diagnostic and T2D group comparisons and interaction effects are shown in Table 2. For reasoning, no main effects of diagnostic group, T2D status, or related interaction effects were found. However, for processing speed and visuospatial memory, main effects of diagnostic group were evident, with inferior performance in BD patients compared to HCs (*Cohen’s d* = −0.03, −0.05). Further, main effects of T2D status were also evident, with poorer performance in patients with T2D compared to those without (*Cohen’s d* = −0.05, −0.02). For visuospatial memory, there was also a significant T2D status by diagnostic group interaction, with follow-up analyses revealing a negative relationship of T2D with performance in the BD group *only (Cohen’s d* = −0.27) (Table 3).

**Table 2:** Associations of T2D with the cognitive domains measured on a continuous scale.

Domain	Comparisons^a^	Group	M^b^	SD	Post-Hoc^c^	d^d^
Main effect of diagnostic group						
Processing speed	**F (1,81805) = 4.56,** ***p*** = 0.033*	BD HC	585.09 575.46	251.47 334.95	BD > HC	−0.03
Visuospatial memory	**F (1, 81269) = 13.33,** ***p*** < 0.001*	BD HC	4.15 3.80	5.93 7.87	BD > HC	−0.05
Reasoning	F (1,79974) = 2.55, *p* = 0.110	BD HC	5.70 5.90	4.56 6.00	----	0.04
Main effect of T2D status						
Processing speed	**F (1,81805) = 12.14,** ***p*** < 0.001*	T2D NT2D	591.64 568.90	375.52 462.33	T2D > No T2D	−0.05
Visuospatial memory	**F (1, 81269) = 7.23,** ***p*** = 0.007*	T2D NT2D	4.10 3.86	8.88 10.96	T2D > No T2D	−0.02
Reasoning	F (1,79974) = 2.19, *p* = 0.139	T2D NT2D	5.59 6.01	6.72 8.17	----	0.06
Diagnostic group* T2D status interaction						
Processing speed	F (1,81805) = 3.19, *p* = 0.074	BD T2D BD NT2D HC T2D HC NT2D	597.21 572.59 585.71 565.21	115.40 123.01 134.82 115.24	----	N/A
Visuospatial memory	**F (1, 81269) = 7.72,** ***p*** = 0.005*	BD T2D BD NT2D HC T2D HC NT2D	4.37 3.94 3.82 3.78	2.73 2.91 3.17 2.72	----	N/A
Reasoning	F (1,79974) = 0.31, *p* = 0.579	BD T2D BD NT2D HC T2D HC NT2D	5.46 5.95 5.71 6.08	2.04 2.17 2.38 2.03	----	N/A
Diagnostic group* T2D status*age interaction						
Processing speed	**F (1,81805) = 6.58,** ***p*** < 0.001*		N/A	N/A	----	N/A
Visuospatial memory	**F (1, 81269) = 4.19,** ***p*** = 0.006*		N/A	N/A	----	N/A
Reasoning	F (1,79974) = 2.58, *p* = 0.052		N/A	N/A	----	N/A

*BD* bipolar disorder, *HC* healthy controls, *T2D* type 2 diabetes, *NT2D* no type 2 diabetes.

^a^Results reported reflect raw values unadjusted for multiple comparisons. Bold values indicate significance before Benjamini-Hochberg FDR correction for multiple comparisons, and those with an * are significant at *p* < 0.05 after Benjamini-Hochberg FDR.

Benjamini-Hochberg FDR correction.

^b^All values are adjusted for age, sex, educational level, townsend deprivation index, and waist circumference.

^c^If post-hoc relationship is not reported, finding was not significant prior or after FDR correction.

^d^d = Cohen’s d effect sizes.

**Table 3:** Associations of T2D with processing speed and visuospatial memory stratified by diagnostic group and in the full sample.

Domain	Comparisons^a^	Group	M^b^	SD	Post-Hoc^c^	d^d^
**Diagnostic group: BD**.						
Main effect of T2D status						
Processing speed	**F (1, 1485) = 5.79,** ***p*** = 0.016*	T2D NT2D	598.16 565.92	130.64 118.20	T2D > No T2D	−0.26
Visuospatial memory	**F (1, 1472) = 7.37,** ***p*** = 0.007*	T2D NT2D	4.65 3.86	3.11 2.78	T2D > No T2D	−0.27
T2D status*age interaction						
Processing speed	**F (1, 1485) = 4.94,** ***p*** = 0.026*					
Visuospatial memory	**F (1, 1472) = 6.51,** ***p*** = 0.011*					
**Diagnostic group: HCs**.						
Main effect of T2D status						
Processing speed	F (1, 80316) = **19.69,** ***p*** < 0.001*	T2D NT2D	585.90 565.34	134.37 115.17	T2D > No T2D	−0.16
Visuospatial memory	F (1, 79793) = 0.06, *p* = 0.811	T2D NT2D	3.82 3.79	3.17 2.72	----	−0.01
T2D status*age interaction						
Processing speed	F (1, 80316) = **12.88,** ***p*** < 0.001*					
Visuospatial memory	F (1, 79793) = 0.11, *p* = 0.739					

*BD* bipolar disorder, *HC* healthy controls, *T2D* type 2 diabetes, *NT2D* no type 2 diabetes.

^a^Results reported reflect raw values unadjusted for multiple comparisons. Bold values indicate significance before Benjamini-Hochberg FDR correction for multiple comparisons, and those with an * are significant at *p* < 0.05 after Benjamini-Hochberg FDR.

^b^All values are adjusted for sex, educational level, townsend deprivation index, and waist circumference.

^c^If post-hoc relationship is not reported, finding was not significant prior or after FDR correction.

^d^d = Cohen’s d effect sizes.

Logistic regression results are reported in Table 4. In the fully adjusted model, diagnostic group was inversely associated with prospective memory performance, such that the odds of a correct prospective memory result on the first attempt differed by a factor of 0.84 in those with BD (95% CI: 0.74, 0.94). T2D status was also inversely associated with prospective memory performance, with the odds of a correct prospective memory result on the first attempt changing by a factor of 0.75 in those with T2D (95% CI: 0.69, 0.81). No significant interaction effects were observed.

**Table 4:** Association of T2D with the dichotomously coded cognitive test (prospective memory).

	B	S.E.	*p*	Exp(B)	95% Lower Bound CI	95% Upper Bound CI
Diagnostic group	−0.18	0.062	0.004*	0.84	0.74	0.94
T2D status	−0.29	0.039	< 0.001*	0.75	0.69	0.81
Diagnostic group*T2D status	−0.20	1.90	0.915	0.82	0.02	33.64
Diagnostic group*T2D status*age	0.009	0.032	0.786	1.009	0.95	1.08

*T2D* type 2 diabetes.

Note that values for covariates are not displayed for brevity. Covariates were entered at block 1, diagnosis and T2D status entered at block 2, and the interaction term entered at

block 3.

*Significant at *p* < 0.05 after Benjamini-Hochberg FDR correction for multiple comparison.

Bold values = significant before Benjamini-Hochberg FDR.

**Fig. 1:**
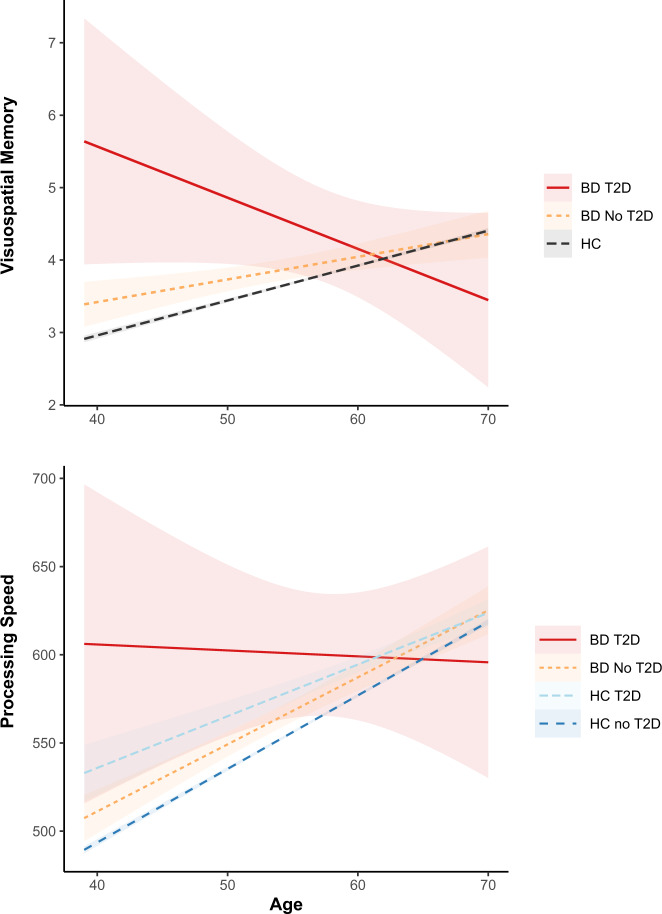
Age-related differences in cognition in BD subgroups with or without T2D v. healthy comparisons. The top graph shows the change in visuospatial memory (reported in the number of errors made) in BD subgroups with (*n* = 73) and without (*n* = 1182) T2D in comparison to healthy comparisons (collapsed as there is no significant difference between those with and without T2D). The bottom graph shows the change in processing speed (reported in milliseconds) in BD subgroups with (*n* = 73) and without (*n* = 1196) T2D in comparison to healthy comparisons with (*n* = 2652) and without (*n* = 63293) T2D. Higher scores indicate worse performance for both visuospatial memory and processing speed.

For processing speed and visuospatial memory, T2D status *by* diagnostic group *by* age interaction effects were also significant (Table 2). Follow-up analyses showed that in the BD group, the interaction of T2D and age was significant for both processing speed and visuospatial memory (Table 3). As indicated in Fig. 1, cognitive test scores were worse in those with BD and T2D versus those without T2D until approximately 60 years of age, whereafter the cognitive performance of the BD group without T2D was worse than those with T2D. In BD patients with comorbid T2D, processing speed scores were equivalent across all participant ages, but visuospatial memory scores were lower in older versus younger patients. In contrast, processing speed and visuospatial memory scores were higher in older versus younger BD patients without T2D. In the HC group, a significant T2D status by age interaction was evident for processing speed but not visuospatial memory (Table 3), where higher processing speed scores were evident in older versus younger HCs both with *and* without T2D (Fig. 1).

### Exploratory medication analyses

BD patients using antidepressants or first-generation antipsychotics had worse processing speed than those who were not; patients using second-generation antipsychotics had worse visuospatial memory than those who were not; and those using sedatives/hypnotic and diabetes medication (both BD and HC) had worse reasoning than those who were not, though effects ranged from negligible to small in size (Tables S1, S2).

## Discussion

In this study, we leveraged a cross-sectional UKB sample to investigate the impact of T2D on cognitive functioning in those with BD compared to a psychiatrically healthy group. In line with our hypotheses, processing speed and prospective memory performance was worse in those with T2D versus those without, irrespective of BD diagnosis. Processing speed, visuospatial memory and prospective memory were also worse in those with BD versus HCs, with the (medium size) effect on visuospatial memory, further exacerbated in patients with BD and T2D. This latter finding suggests an interactive effect of T2D and BD on visuospatial memory, supported by research linking T2D and general cognitive impairment in a *BD-only* sample [32]. It also accords with meta-analytic findings indicating a more general association of visual memory performance with T2D [43]. The mechanistic basis for this relationship of T2D with visuospatial memory performance specifically is not clear.

Our findings also indicated that age-related visuospatial memory and processing speed effects were moderated by comorbid T2D in BD. In those with BD *and* T2D, deficits in these cognitive domains were evident irrespective of age, with performance seen to be either equivalent irrespective of participant age (processing speed), or incrementally improving (visuospatial memory) as a function of increasing participant age. In BD patients *without* T2D, performance on both domains was worse than HCs, however, the age-related slopes of scores remained broadly equivalent. Notably, worse processing speed and visuospatial memory in those with BD *and* T2D were evident at the beginning of the age range indexed (~40 years). Since group differences were of the largest magnitude just prior to the fifth decade of life, the initial cognitive decrement in this group appears to have occurred prior to the midlife period (i.e., <40 years). This pattern of findings suggests that those with comorbid BD and T2D may experience an initial and premature ‘decline’ in cognition relative to that expected on the basis of age. However, the magnitude of deficits resulting from any such decline may lessen as they get older, due to an apparent deviation from a typical age-related trajectory of *ongoing* cognitive deterioration.

That premature cognitive dysfunction was apparent for processing speed in particular, is consistent with several meta-analyses indicating this domain as one of the earliest and most commonly affected in people with T2D [22, 23]. Our finding that HCs *with* T2D also had worse processing speed earlier in the lifespan compared to HCs *without* T2D accords with this, and further implicates T2D, or factors related to it, in the putatively premature decline in cognition seen here in those with BD. To this point, and in light of our pattern of findings, it is possible that certain lifestyle or genetic factors linked to T2D and BD may catalyse cognitive impairments in this group initially, while other factors unmeasured in the current study but of relevance to this group specifically, may somehow offset *ongoing* cognitive decline. For example, emerging evidence indicates a protective role of diet quality in the context of cognitive performance and cognitive ageing [21, 44–47], and given the importance of diet in diabetes control *and* its role in improving depressive symptoms [48, 49], it is possible that BD patients with T2D have particularly strict diets. This is purely speculative however, and not based on data that we have analysed in the current sample. Future work would do well to explore this possibility further.

There are likely to be multiple pathways by which T2D interacts with BD to impact cognition. For example, a recent UKB study indicated that the T2D-cognition relationship was partially mediated by macrovascular problems, depressive symptoms, and visceral obesity [50], which are prevalent in BD and potentially represent underlying mechanisms [51]. Other biological pathways include oxidative stress, mitochondrial dysfunction, neuroinflammation, and HPA axis dysregulation [18, 52], which are common to BD [10] and can be catalysed, in part, by the abnormal insulin signalling, central insulin resistance, or hyperglycaemic excursions associated with T2D [18, 52, 53]. Mechanistically, these factors may result in neuronal damage, neurodegeneration, and declines in both neurogenesis and neuroplasticity [52], all of which are associated with cognitive dysfunction, including both processing speed and memory specifically [54]. The mediating effect of depressive symptoms is particularly relevant, as depressive symptoms have been consistently linked to cognitive impairment in T2D [55–57]. This raises the question as to whether the findings observed here are *specific* to BD or relate to mood disorders more generally.

To our knowledge, this is the largest study to have examined the T2D-cognition relationship in BD. It is also the first BD study to include a direct and validated measure of T2D as well as an analysis of age-related associations [58, 59]. Other strengths relate to the comprehensiveness of demographic and lifestyle variables available in the UKB, which allowed us to control for relevant factors more extensively than in previous research. This includes analyses of psychotropic and diabetes medication use, which was revealed to have quite limited effects on the variables of interest. One exception to this was antidepressant use, which was more prevalent in the BD group with comorbid T2D and was marginally associated with processing speed in the BD sample as a whole. It is possible that patients with a more severe illness were using antidepressants, such that the worse processing speed reflects an outcome of greater  disease severity. To this end, more participants with BD and T2D were using sedatives than BD participants without T2D (7.1 versus 3.6%). While this difference was not significant, it remains possible that sedative use had an effect on the findings, although it is unlikely to have driven the results given the small percentage of participants from the total BD and T2D group using this medication. Further research should aim to examine dosage information and prior medication history to determine if psychotropic medication use is of relevance.

Several limitations should also be considered. Firstly, the study was cross-sectional, which limited our capacity to identify temporal changes in cognition over time. Nonetheless, by modelling age in the data, our preliminary findings raise new hypotheses about the trajectory of cognition in BD as it relates to T2D, which can justify future longitudinal work in this area that is substantially more costly and difficult to do. Second, we modelled the data in a linear fashion only, which may have hidden certain age-relevant effects that may only be apparent with non-linear analyses. Further, an algorithm was used to determine T2D status, which relies in part, on self-reported data. This may result in measurement error, although there is evidence showing the algorithm has reasonable accuracy (96%) and is externally valid [38]. Similarly, BD status was determined using a criterion established for the UKB, rather than using a validated diagnostic measure. The criterion, however, was based on symptoms within the DSM-IV and has shown promising validity [35]. Moreover, the lack of detailed mental health data meant we were unable to control for current mood state. Further research with confirmed diagnoses and symptom data is warranted.

Finally, the prevalence of T2D was lower than what is generally reported in the literature (5.6% compared to 11% in BD, 4.2% compared to 7.9% in the general population), and relatively well controlled (average HbA1c of 51 mmol/mol**)**. Individuals with mental health disorders in the UKB also appear to be more highly functioning than usual [60], as exemplified by the small proportion of BD patients using psychotropic medication in our study (30.8%). The magnitude of cognitive deficits was also lower than typically seen in BD and T2D studies, which is likely to relate to the sensitivity of the cognitive tests available in the UK Biobank (they had not been validated), and the limited number of domains assessed. That associations were still evident despite these limitations, however, does suggest that more extensive deficits are likely to be apparent in studies with more comprehensive cognitive assessments and in samples more reflective of BD or T2D generally. Relevantly, deficits of somewhat larger effect were evident (Cohen’s d of −0.16 to −0.27, comparable to *some* past research [43]) when the sample was stratified by diagnosis compared to effects seen in the T2D-non-T2D comparisons alone, which further highlights the clinical meaningfulness of comorbid T2D as a risk factor for cognitive dysfunction in BD.

In sum, our findings from this large UKB cohort suggest that BD and T2D may interactively influence cognitive performance deficits in processing speed, and prospective and visuospatial memory across the mid-life period. Effects of T2D on processing speed and prospective memory are also apparent in the HC group, albeit to a lesser extent. Our findings accord with evidence indicating clinical course, psychosocial functioning, and health service utilization in BD are disproportionately impacted by the burden of medical comorbidities [61, 62]. They are also consistent with a small but growing literature on BD implicating metabolic syndrome components and obesity in more severe cognitive and detrimental brain imaging outcomes [63]. Together, these findings open up the possibility of targeting elements of the associated biological pathways, such as insulin, therapeutically. Intranasal insulin treatment has been linked with cognitive improvement in BD [64], and more recently, preliminary evidence has described pro-cognitive effects of metformin use in those with a mood disorder [65]. These treatments, alongside other treatments targeting insulin signalling (such as dulaglutide which has cognitive preserving effects in T2D [66], or non-pharmacological metabolic treatments) might thus be considered as possible intervention agents in clinical trials for cognitive enhancement in BD.

Notably, we found that the magnitude of cognitive deficits in those with BD *and* T2D was largest during the fifth decade of life. This may reflect the occurrence of an early and premature deterioration of cognitive function in BD on account of T2D, consistent with evidence of premature ageing in other biological systems [67]. However, since the magnitude of effects reduced as normal age-related performance decrements in the psychiatrically healthy group increased, our findings raise the possibility that cognitive dysfunction in those with comorbid BD and T2D is not pathologically progressive. Clinically, these findings indicate the importance of considering BD, T2D, and age *together* when assessing patients with these disorders. Further longitudinal studies are needed to examine this explicitly and to identify whether there are protective factors in those with comorbid BD and T2D that offset ongoing decline in cognition with age. These studies will have important implications for the care of this patient group in the clinic and will help to elucidate the specific pathways that are mechanistically involved in the adverse cognitive outcomes arising from the comorbidity of BD and T2D.

## Supplementary information

Supplementary Material

Table S1
