# Supplementary material for: Interactive relationships of Type 2 diabetes and bipolar disorder with cognition: evidence of putative premature cognitive ageing in the UK Biobank Cohort
Source: Neuropsychopharmacology. 2022 Oct 15;48(2):362–70. doi: 10.1038/s41386-022-01471-6 (PMC9750982; doi:10.1038/s41386-022-01471-6)
Supplement: Supplementary file 1 — Supplementary Material [file 41386_2022_1471_MOESM1_ESM.doc]

**SUPPLEMENTARY MATERIAL**

**Synergistic Relationships of Type 2 Diabetes and Bipolar Disorder with Cognition: Evidence of Putative Premature Cognitive Ageing in the UK Biobank Cohort**

**Table of Contents**

[**Supplementary Methods** 2](#__RefHeading___Toc112919659)

[**Supplementary Tables** 3](#__RefHeading___Toc112919660)

[**Table S1**. Associations between medication use and cognitive domains measured on a continuous scale 3](#__RefHeading___Toc112919661)

[**Table S2***.* Association between medication use and dichotomously coded cognitive test (prospective memory) 6](#__RefHeading___Toc112919662)

[**Supplementary Figures** 7](#__RefHeading___Toc112919663)

[**FIGURE S1.** Flow chart of participant sampling 7](#__RefHeading___Toc112919664)

[**Supplementary Appendices** 8](#__RefHeading___Toc112919665)

[**Appendix S1.** Excluded neurological conditions. 8](#__RefHeading___Toc112919666)

[**Appendix S2.** List of diabetes medications included in dichotomous diabetes medication variable. 8](#__RefHeading___Toc112919667)

[**Appendix S3.** List of psychotropic medications 9](#__RefHeading___Toc112919668)

[**References** 11](#__RefHeading___Toc112919669)

# **Supplementary Methods**

**International Physical Activity Questionnaire**

Physical activity was measured using adapted questions from the International Physical Activity Questionnaire (IPAQ) short form. This questionnaire utilises six self-report questions to assess the frequency, intensity, and duration of walking, moderate-intensity, and vigorous-intensity physical activity (Craig et al., 2003). Time spent in each activity (walking, moderate, vigorous activities) were weighted by the energy expenditure for each activity, which was then used to calculate metabolic equivalent of task (MET) minutes / week, a continuous measure of weekly physical activity. Participants who recorded “unable to walk” were excluded from the analyses (n=90). Additionally, the responses “do not know” and “prefer not to answer” were recoded as missing data, and thus not included in any analyses.

**Data cleaning and additional analyses**

Initial data cleaning revealed outliers for several key variables (visuo-spatial memory, processing speed, waist circumference and SES), so sensitivity analyses were run with the outliers removed. Removing visuospatial memory outliers (scores ≥ 14) significantly altered results, and thus outliers were removed for the final analysis and are the sole findings reported. Removal of outliers for processing speed, waist circumference, and SES did not significantly alter results, and thus outliers were left in for the final analyses.

Additional analyses were run accounting for insulin use, HbA1c levels, physical activity, and smoking status, due to known influences of the latter factors on cognition and because those with T2D on insulin treatment have more severe T2D. Analyses were re-run four times; (1) excluding participants taking insulin (n= 776 HC, n= 21 BD excluded), (2) excluding those with high HbA1c levels (possibly reflecting insulin resistance without a T2D diagnosis) from the no T2D group (n= 3104 HC, n= 57 BD excluded), (3) including a continuous physical activity variable (see above) as a covariate, and (4) including smoking status (yes/no) as a covariate. Results were largely unchanged, and thus the findings are not presented for brevity.

**Exploratory medication analyses**

Given the possibility of confounding effects of psychotropic medication and diabetes medication on cognition, additional analyses were run to determine whether group differences in cognitive scores were evident between medicated and un-medicated individuals in the BD and T2D (inclusive of those with BD) subsamples (subsamples were analysed separately). Psychotropic medications were grouped into medication classes (see below appendix S2) based on previous research (Cullen et al., 2015). As a result, mood stabilisers and anticonvulsants were grouped together. Multiple univariate ANCOVAs were run for the continuous cognitive variables, and a logistic regression for prospective memory, with psychotropic medications, and diabetes medication as the respective independent variables. Covariates remained the same as those used in the primary analyses.

# **Supplementary Tables**

| **Table S1**. Associations between medication use and cognitive domains measured on a continuous scale | | | | | | |  | |
| --- | --- | --- | --- | --- | --- | --- | --- | --- |
| **Domain** | **Comparisons^a^** | **Group** | **M^b^** | **SD** | **Post-Hoc^c^** | **d^d^** | |  |
| Main effect of mood stabilisers |  |  |  |  |  |  | |  |
| Processing speed | F (1,1482) = 1.01, p = 0.315 | NU  U | 604.11  614.68 | 556.92  211.03 | ---- | 0.02 | |  |
| Visuospatial memory | F (1,1469) = 1.34, p = 0.248 | NU  U | 4.24  4.52 | 13.27  5.00 | ---- | 0.03 | |  |
| Reasoning | **F (1,1440) = 4.23, p = 0.040** | NU  U | 5.53  5.91 | 10.71  3.99 | NU < U | 0.05 | |  |
| Main effect of antidepressants |  |  |  |  |  |  | |  |
| Processing speed | **F (1,1482) = 25.40, p < 0.001*** | NU  U | 590.01  629.15 | 520.89  269.86 | ---- | 0.09 | |  |
| Visuospatial memory | F (1,1469) = 0.11, p = 0.742 | NU  U | 4.35  4.41 | 12.31  6.38 | ---- | 0.01 | |  |
| Reasoning | F (1,1440) = 0.29, p = 0.592 | NU  U | 5.76  5.68 | 9.96  5.14 | NU > U | -0.01 | |  |
| Main effect of first-generation APs |  |  |  |  |  |  | |  |
| Processing speed | **F (1,1482) = 9.60, p = 0.002*** | NU  U | 572.75  646.41 | 360.64  124.87 | NU < U | 0.27 | |  |
| Visuospatial memory | F (1,1469) = 0.55, p = 0.457 | NU  U | 4.17  4.59 | 8.75  2.97 | ---- | 0.06 | |  |
| Reasoning | F (1,1440) = 0.94, p = 0.333 | NU  U | 5.59  5.94 | 6.69  2.24 | ---- | 0.09 | |  |
| Main effect of second-generation APs |  |  |  |  |  |  | |  |
| Processing speed | F (1,1482) = 0.02, p = 0.891 | N  U | 608.63  610.53 | 530.49  167.61 | ---- | 0.00 | |  |
| Visuospatial memory | **F (1,1469) = 8.58, p = 0.003*** | NU  U | 3.89  4.86 | 12.66  3.97 | NU < U | 0.10 | |  |
| Reasoning | F (1,1440) = 0.29, p = 0.593 | NU  U | 5.79  5.65 | 10.13  3.16 | ---- | -0.02 | |  |
| Main effect of sedatives / hypnotics |  |  |  |  |  |  | |  |
| Processing speed | F (1,1482) = 1.45, p = 0.229 | NU  U | 619.31  599.85 | 505.78  147.67 | ---- | -0.05 | |  |
| Visuospatial memory | F (1,1469) = 2.10, p = 0.147 | NU  U | 4.67  4.09 | 12.01  3.45 | ---- | -0.07 | |  |
| Reasoning | **F (1,1440) = 6.84, p = 0.009*** | NU  U | 6.12  5.32 | 9.65  2.70 | NU > U | -0.11 | |  |
| Main effect of diabetes medication |  |  |  |  |  |  | |  |
| Processing speed | F (1,3424) = 0.16, p = 0.694 | NU  U | 598.43  596.50 | 132.06  131.72 | ---- | -0.01 | |  |
| Visuospatial memory | F (1, 3417) = 0.10, p = 0.750 | NU  U | 4.01  3.97 | 2.87  2.86 | ---- | -0.01 | |  |
| Reasoning | **F (1,3237) = 7.21, p = 0.007*** | NU  U | 5.72  5.51 | 2.12  2.11 | NU > U | -0.10 | |  |

NU = Non-users, U = users, APs = antipsychotics.

Note that psychotropic medications were analysed in the BD sample only, and diabetes medication in the T2D sample only.

^a^Results reported reflect raw values unadjusted for multiple comparisons. Bold values indicate significance before Benjamini-Hochberg FDR correction for multiple comparisons, and those with an * are significant at p <.05 after Benjamini-Hochberg FDR.

^b^All values are adjusted for age, sex, educational level, townsend deprivation index, and body mass index.

^c^If post-hoc relationship is not reported, finding was not significant prior or after FDR correction.

^d^d = Cohen’s d effect sizes.

*Significant at p < .05 after Benjamini-Hochberg FDR correction for multiple comparisons.

Bold values = significant before Benjamini-Hochberg FDR.

| **Table S2***.* Association between medication use and dichotomously coded cognitive test (prospective memory) | | | | | | | |
| --- | --- | --- | --- | --- | --- | --- | --- |
|  | B | S.E. | *p* | Exp(B) | 95%  Lower Bound CI | 95%  Upper Bound CI |  |
| Mood stabilisers | -0.17 | 0.20 | 0.375 | 0.84 | 0.57 | 1.23 |  |
| Antidepressants | -0.18 | 0.15 | 0.218 | 0.83 | 0.62 | 1.11 |  |
| First-generation APs | 0.06 | 0.45 | 0.896 | 1.06 | 0.44 | 2.57 |  |
| Second-generation APs | -0.59 | 0.25 | **0.020** | 0.55 | 0.34 | 0.91 |  |
| Sedatives / hypnotics | -0.44 | 0.29 | 0.133 | 0.65 | 0.37 | 1.14 |  |
| Diabetes medication | -0.14 | 0.083 | 0.101 | 0.87 | 0.75 | 1.03 |  |

APs = antipsychotics

Note that psychotropic medications were analysed in the BD sample only, and diabetes medication in the T2D sample only. Values for covariates are not displayed for brevity.

Covariates were entered at block 1, diagnosis and medication use entered at block 2, and the

interaction term entered at block 3.

Bold values = significant before Benjamini-Hochberg FDR.

# **Supplementary Figures**

**
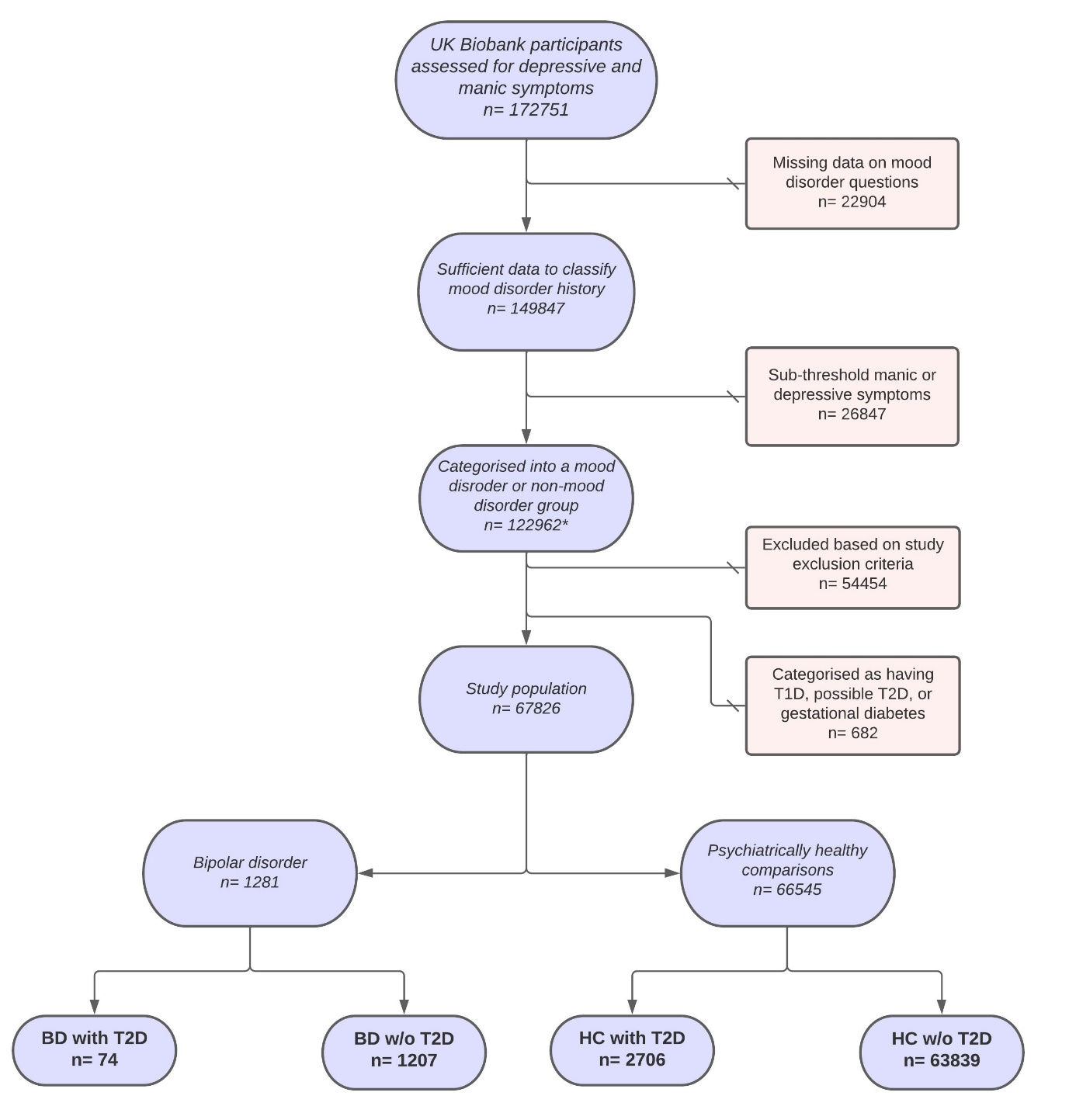
**

**FIGURE S1.** Flow chart of participant sampling^a^.

^a^A number of participants asked for their data to be removed from the UK Biobank database following the original mood disorder categorisations (n= 38) and were removed from the flowchart where the asterix is placed.

# **Supplementary Appendices**

**Appendix S1.** Excluded neurological conditions. Self-reported by participants; from data fields 6150, 20001 and 20002.

- Brain cancer/primary malignant tumour

- Brain haemorrhage

- Brain/intracranial abscess

- Cerebral aneurysm

- Cerebral palsy

- Chronic/degenerative neurological problem

- Dementia/Alzheimer's disease/cognitive impairment

- Encephalitis

- Epilepsy

- Head injury

- Infection of nervous system

- Ischaemic stroke

- Meningeal cancer/malignant meningioma

- Meningioma (benign)

- Meningitis

- Motor neurone disease

- Multiple sclerosis

- Neurological injury/trauma

- Neuroma (benign)

- Other demyelinating condition

- Other neurological problem

- Parkinson's disease

- Spina bifida

- Stroke

- Subarachnoid haemorrhage

- Subdural haematoma

- Transient ischaemic attack

**Appendix S2.** List of diabetes medications included in dichotomous diabetes medication variable. Recorded by nurse in interview with participant, data field 20003.

- Insulin

- Metformin

- Sulfonylurea

- Arcabose

- Glucotard

- Meglitinide

- Glitazone

- Non-metformin oral anti-diabetic drugs.

**Appendix S3. List of psychotropic medications (recorded by nurse in interview with participant; data field 20003).**

| **Mood Stabiliser** | **Selective-serotonin reuptake inhibitor (SSRIs)^β^** | **Other anti-depressants^β^** | **Typical antipsychotics** | **Atypical antipsychotics** | **Sedatives/hypnotics** |
| --- | --- | --- | --- | --- | --- |
| lithium product  Priadel (lithium)  Camcolit (lithium)  sodium valproate  Epilim (sodium valproate)  Depakote (semisodium valproate)  valproic acid  carbamazepine product  carbamazepine  Tegretol (carbamazepine)  Teril (carbamazepine)  Teril retard (carbamazepine)  Timonil retard (carbamazepine)  Epimaz (carbamazepine) | paroxetine  Seroxat (paroxetine)  fluoxetine  Prozac (fluoxetine)  citalopram  Cipramil (citalopram)  escitalopram  Cipralex (escitalopram)  sertraline  Lustral (sertraline)  fluvoxamine | mirtazapine  Zispin (mirtazapine)  duloxetine  Cymbalta (duloxetine)  Yentreve (duloxetine)  venlafaxine  Efexor (venlafaxine)  amitriptyline  Elavil (amitriptyline)  Tryptizol (amitriptyline)  Lentizol (amitriptyline)  amitriptyline+perphenazine  Triptafen (amitriptyline+perphenazine)  amitriptyline+chlordiazepoxide  Limbitrol 10 (amitriptyline+chlordiazepoxide)  Limbitrol-5 (amitriptyline+chlordiazepoxide)  phenelzine  maoi - phenelzine  Nardil (phenelzine)  moclobemide  Manerix (moclobemide)  imipramine  Tofranil (imipramine)  trimipramine  Surmontil (trimipramine)  dothiepin  dosulepin  Prothiaden (dosulepin)  Thaden (dosulepin)  clomipramine  Anafranil (clomipramine)  lofepramine  Gamanil (lofepramine)  Lomont (lofepramine)  mianserin  Bolvidon (mianserin)  Norval (mianserin) | chlorpromazine  cpz - chlorpromazine  Largactil (chlorpromazine)  haloperidol  Haldol (haloperidol)  Serenace (haloperidol)  fluphenazine decanoate  fluphenazine  Modecate (fluphenazine)  Moditen tablet (fluphenazine)  Moditen enanthate (fluphenazine)  flupentixol  Flupenthixol (flupentixol)  Depixol (flupentixol)  Fluanxol (flupentixol)  zuclopenthixol  Clopixol (zuclopenthixol)  loxapine  Loxapac (loxapine)  droperidol  Droleptan (droperidol)  trifluoperazine  Stelazine (trifluoperazine)  thioridazine  Melleril (thioridazine) | quetiapine  Seroquel (quetiapine)  risperidone  Risperdal (risperidone)  olanzapine  Zyprexa (olanzapine)  aripiprazole  Abilify (aripiprazole)  amisulpride  Solian (amisulpride)  clozapine  Clozaril (clozapine | diazepam  diazepam product  Valium tablet (diazepam)  Valium syrup (diazepam)  Valium supp (diazepam)  temazepam  Normison (temazepam)  Euhypnos (temazepam)  zopiclone  Zimovane (zopiclone)  zaleplon  Sonata (zaleplon)  zolpidem  Stilnoct (zolpidem)  nitrazepam  Mogadon (nitrazepam)  Nitrados (nitrazepam)  Remnos (nitrazepam)  Somnite (nitrazepam)  Noctesed (nitrazepam)  Surem (nitrazepam)  Unisomnia (nitrazepam)  flunitrazepam  Rohypnol (flunitrazepam)  triazolam  Halcion (triazolam |

β In the current study, SSRIs and other antidepressants were combined to create one variable titled “antidepressants”.

# **References**

Craig, C. L., Marshall, A. L., Sjöström, M., Bauman, A. E., Booth, M. L., Ainsworth, B. E., … Oja, P. (2003). International physical activity questionnaire: 12-Country reliability and validity. *Medicine and Science in Sports and Exercise*. https://doi.org/10.1249/01.MSS.0000078924.61453.FB

Cullen, B., Nicholl, B. I., Mackay, D. F., Martin, D., Ul-Haq, Z., McIntosh, A., … Smith, D. J. (2015). Cognitive function and lifetime features of depression and bipolar disorder in a large population sample: Cross-sectional study of 143,828 UK Biobank participants. *European Psychiatry*, *30*(8), 950–958. https://doi.org/10.1016/j.eurpsy.2015.08.006
